# Supplementary material for: Symmetrical arrangement of positively charged residues around the 5-fold axes of SAT type foot-and-mouth disease virus enhances cell culture of field viruses
Source: PLoS Pathog. 2020 Sep 29;16(9):e1008828. doi: 10.1371/journal.ppat.1008828 (PMC7577442; doi:10.1371/journal.ppat.1008828)
Supplement: S1 Table — (DOC) [file ppat.1008828.s001.doc]

**S1 TABLE**

**S1Table:** Summary of the amino acid substitutions in the outer capsid proteins of SAT1 and SAT2 viruses resulting from cytolytic passages in BHK-21 cells

| **Protein** | **Structure**  **element** | **SAT1 isolate^a*^** | | | | | | | | | |  |
| --- | --- | --- | --- | --- | --- | --- | --- | --- | --- | --- | --- | --- |
|  |  | **KNP/148/91** | **KNP/41/95** | **ZIM/13/90** | **KEN/5/98** | **TAN/1/99** | **UGA/1/97** | **SUD/3/76** | **NIG/5/81** | **NIG/15/75** | **NIG/6/76** | **NAM/307/98^b^** |
| **VP2** | **βB-βC** | -  **Q2074R**  -  D2134E  S2196N | -  -  -  -  - | -  -  -  -  - | D2039A  -  -  -  - | -  -  -  -  - | -  -  L2115Q  **E2133K**  - | -  -  V2090I  -  - | -  -  -  -  - | -  -  -  -  - | -  -  A2107V  -  - | -  -  -  -  - |
| **VP3** | **βE-βF**  **βG-βH** | D3009N  T3043N  F3044L  N3131H  N3135E  -  -  - | -  -  -  H3131S  -  -  -  - | -  -  -  R3129G  -  -  -  - | -  -  -  -  -  -  -  R3220H | N3013G M3029V  -  -  -  -  -  - | -  -  -  -  -  -  -  - | A3006G  -  -  -  -  -  -  - | -  -  -  -  -  -  -  - | -  -  -  -  -  -  -  - | -  -  -  -  -  -  -  - | -  -  -  -  **E3135K**  **E3175K**  S3203T  S3219L |
| **VP1** | **βD-βE**  **βF-βG**  **βG-βH** | -  -  R1049K  -  -  -  -  -  -  **G1112R**  -  -  V1179E K1206R  K1210R  -  - | -  -  -  -  -  **E1084K**  **-**  **-**  **N1111K**  -  -  -  -  -  S1212A  -  - | **-**  **-**  **-**  **-**  -  -  K1086Q  -  **N1111K G1112R**  -  -  -  -  -  -  - | -  -  -  -  -  **E1084K**  **W1087R** | **-**  **-**  **-**  **-**  **-**  **-**  **-**  **-**  **-**  **E1112K**  **-**  S1141C **D1181N**  -  -  -  - | -  -  H1046N  -  **E1058K**  -  -  -  -  -  V1127L  -  -  -  -  -  - | **C1026R**  **-**  -  -  -  -  -  -  **N111K**  **G112R**  -  -  -  -  -  -  - | **-**  **-**  **N1048K**  **-**  **-**  **-**  **-**  **-**  **N1111K**  **-**  **-**  **-**  H1183Y  -  -  -  - | **-**  **-**  **-**  **-**  **-**  **-**  **-**  **-**  **N1111K**  -  -  -  **D1180N**  -  -  -  - | -  -  -  -  A1061V  -  -  -  **N1111K**  -  -  -  -  -  -  -  - | T1025A  A1033T  -  -  -  -  -  -  -  G1112D  -  -  -  -  -  G1157A  G1177Q |

**S1Table:** Continues ….

| **Protein** | **Structure**  **element** | **SAT2 isolate^a*^** | | | |  |
| --- | --- | --- | --- | --- | --- | --- |
|  |  | **KNP/2/89** | **KNP/51/93** | **ZIM/10/91** | **UGA/2/02** | **SAU/6/00** |
| **VP2** | **βC-βD** | -  -  K2128E  - | -  -  -  - | -  -  -  - | L2147F  T2158I  F2191L | T2099A  -  - |
| **VP3** |  | C3007W | V3188I |  | S3120F | D3193N |
| **VP1** | **βB-βC**  **βD-βE**  **βF-βG**  **βG-βH** | S1050N,  -  **D1083N Q1085R**  -  -  - | **-**  **-**  **E1083K**  -  **D1110G**  **-**  - | -  -  -  -  N1110Q  -  - | R1045G,  -  **E1083K**  -  -  -  R1189C | **V1050L**  **D1055N**  -  **-**  **T1158K**  **-** |

^*^Amino acid changes to a positive charge in surface exposed loops are shown in bold-italics. The loss of a negative charge is indicated in bold.

^a^ The amino acid residues have been numbered independently for each protein. For each residue, the first digit indicates the protein (VP1, VP2 or VP3) and the last three digits the amino acid position in either a SAT1 or SAT2 alignment. The P1 polypeptide of SAT1 viruses is 744 amino acids and that of SAT2 viruses 741 amino acids.

^b^ FMDV SAT1/NAM/307/98 amino acid changes published in Maree *et al*., 2010 (1).

**References**

1. Maree FF, Blignaut B, de Beer TAP, Visser N, Rieder EA. 2010. Mapping of amino acid residues responsible for adhesion of cell culture-adapted foot-and-mouth disease SAT type viruses. Virus Res 153:82–91.
