# Supplementary material for: Symmetrical arrangement of positively charged residues around the 5-fold axes of SAT type foot-and-mouth disease virus enhances cell culture of field viruses
Source: PLoS Pathog. 2020 Sep 29;16(9):e1008828. doi: 10.1371/journal.ppat.1008828 (PMC7577442; doi:10.1371/journal.ppat.1008828)
Supplement: S2 Table — (DOC) [file ppat.1008828.s002.doc]

**S2 TABLE**

**Cross-reactivity and predicted protection against heterologous field isolates**

A SAT2 isolate causing a severe outbreak in dairy herds in Saudi Arabia in 2000, *i.e.* SAT2/SAU/6/00 was used to measure cross-reactivity in one-way antigenic relationships. One SAT2 isolate, SAU/6/00 calf thyroid (CT) passage of one (SAT2/SAU/6/00^CT1^), was passaged 58 times in BHK-21 suspension cells (designated SAT2/SAU/6/00^BHK58^). The differences in plaque morphology on BHK-21 cells of the SAT2/SAU/6/00^CT1^ and SAT2/SAU/6/00^BHK58^ was described in the main paper.

We applied one-way antigenic relationships (r_1_-values), determined from VNT data, to measure potential antigenic drift of cell culture-passaged or the mutated v^SAU^SAT2 viruses. Cattle sera were prepared by two consecutive vaccinations of two groups of three cattle using vaccines prepared from SAT2/SAU/6/00^CT1^ and SAT2/SAU/6/00^BHK58^ virus seed stocks. The sera were inactivated at 56°C for 30 min before the sera from each vaccinated group of animals was pooled and used in subsequent virus neutralization assays. One-way antigenic relationships (r_1_-values) were calculated as the ratio between the derivate of SAT2/SAU/6/00 and the homologous (SAT2/SAU/6/00^CT1^ or SAT2/SAU/6/00^BHK58^) serum titers. The r_1_-value is an indication of the antigenic cross-reactivity between viruses and is interpreted as values ≥0.30 meaning antigenic similarity or values <0.30 as antigenically different.

Bovine sera from animal vaccinated with SAT2/SAU/6/00^BHK58^ reacted similarly against SAT2/SAU/6/00^CT1^, v^SAU^SAT2 and five mutant chimeric viruses (S2 Table). Correspondingly, SAT2/SAU/6/00^CT1^ vaccinated cattle sera showed r_1_-values that range from 0.89 to 1.2 against SAT2/SAU/6/00^BHK58^, v^SAU^SAT2 and the five mutant chimeric viruses (S2 Table). Therefore is can be concluded that based on the cross neutralization data, the antigenic features of SAT2/SAU/6/00 seem to be unchanged following 58 passages on BHK-21 cells or the introduction of amino acids on the structurally exposed loops of the VP1 protein.

**S2 Table**: Virus neutralization and predicted cross-reactivity of SAT2/SAU/6/00 vaccinated cattle sera to the isolates and chimeric mutant viruses.

| Virus | **SAT2/SAU/6/00 cattle s**er**a** | | | |
| --- | --- | --- | --- | --- |
|  | **CT1** | | **BHK58** | |
|  | VNT^1^ | r_1_-value^2^ | VNT^1^ | r_1_-value^2^ |
| **ZIM/6/00^CT1^**  **ZIM/6/00^BHK58^**  **v^SAU^SAT2**  **rv^SAU^VP3^158K^**  **rv^SAU^VP1^50L,55N^ rv^SAU^VP1^158K^**  **rv^SAU^VP1^83K^**  **rv^SAU^VP1^83K,85R^**  **rv^SAU^VP1^110KRR^** | 2.7  2.9  2.7  nd  2.9  2.8  2.7  2.6  2.7 | 1.0  1.2  1.0  nd  1.2  1.1  0.94  0.89  0.93 | 3.2  3.1  3.1  nd  3.0  3.1  3.1  3.0  3.1 | 1.1  1.0  1.0  nd  0.94  1.0  0.97  0.92  1.0 |

^1^The virus neutralization titres are expressed as log_10_ reciprocal antibody dilution required for 50% neutralization of 100 tissue culture infectious units. The mean values of two repeats are depicted. Nd=not done

^2^The r_1_-values were calculated as the ratio between the heterologous and homologous serum titres and were interpreted as proposed by OIE Manual (2018). r_1_-values ≥0.3 were considered to sufficient cross-reactive.
