# Supplementary material for: Symmetrical arrangement of positively charged residues around the 5-fold axes of SAT type foot-and-mouth disease virus enhances cell culture of field viruses
Source: PLoS Pathog. 2020 Sep 29;16(9):e1008828. doi: 10.1371/journal.ppat.1008828 (PMC7577442; doi:10.1371/journal.ppat.1008828)
Supplement: Attachment — Submitted filename: Response to Reviewers_250520.docx [file ppat.1008828.s003.docx]

**Agricultural Research Council**

**Onderstepoort Veterinary Research Institute**

**Transboundary Animal Diseases Programme**

**Old Soutpan Road**

**Onderstepoort, 0110**

**South Africa**

**Tel. +27 12 529 9575**

**Fax. +27 12 529 9505**

27 May 2020

To: The Associate Editor, Section Editor and Editor-in-Chiefs– PLoS Pathogens Journal

**Ref: PPATHOGENS-D-20-00242 -** “**Symmetrical arrangement of positively charged residues around the 5-fold axes of SAT type foot-and-mouth disease virus enhances cell culture of field viruses”**

Thank you for communicating to us the expert reviewer’s comments of our manuscript by Chitray et al., with reference number PPATHOGENS-D-20-00242. All authors are in agreement with the revised manuscript.

We found the comments of the reviewers very useful, and we have addressed all of the comments from the reviewers accordingly (point-by-point list enclosed). The first reviewer indicated “the molecular virology experiments are thoroughly designed, and the results are rich and convincing.” The second and third reviewers stated that “this is a meticulous study by well-respected authors which has generated robust data” and “the data hangs together well.” The reviewers’ recommendations were valid and thus, we have revised the manuscript to include additions to the text to improve clarity, readability and shortcomings. The changes are tracked in the enclosed revised document.

We hope that the revised manuscript may now be acceptable for publication in the PLoS Pathogens Journal.

Sincerely yours,

Francois F Maree, PhD

Specialist Scientist

Agricultural Research Council

Onderstepoort Veterinary Research Institute

Vaccines and Diagnostic Development

**Response to Reviewers**

- **Part I – Summary**

**Reviewer #1:** FMDVs remain as significant threats to poultry and livestock industry. Developing vaccines from emerging new strains are difficult due to that some of the field viruses are unable to be adapted to cell cultures. This study analyzed the mutations of cell-culture-adapted field viruses. Many of these mutations are positively charged residues clustered around the icosahedral five-fold axis. The authors then designed a series of experiments to show that these mutated viruses have adapted new host receptors, heparin sulfate proteoglycan (HSPG). They also generated chimera viruses that mimic those observed mutations. These chimera viruses were able to adapt to cell culture well, which provide a novel way to quickly adapt emerging field viruses to cell culture for vaccine developments.

The molecular virology experiments are thoroughly designed, and the results are rich and convincing. Many different aspects have been tested and discussed. This part is the strength of the paper.

The molecular simulation docking is OK but not as convincing as the other experiments. With highly positively charged 5-fold, the negatively charged heparin will be docked there. The most convincing results will be a structure of the virus/heparin complex. With current cryo-EM techniques, obtain a complex structure using purified virus and heparin can be done very quickly. No need to obtain atomic resolution. Extra densities on the five-fold positively charged areas will prove the virus has adapted heparin as the receptor if using purified virus incubate with heparin. The author team has an expert in using cryo-EM to solve picornavirus structures.

In summary, the paper is a very good one with a lot of convincing data from lab experiments. The simulation part is not necessary. Completely remove the simulation will not affect the significance of the discovery. Normally simulation can provide useful hints or suggestions for designing lab experiments. Here, the reviewer does not see that. Simulation itself cannot be a strong data to prove the receptor binding.

**Author’s comment**: We agree with the reviewer that a cryo-EM structure of purified virus/heparin complex would indeed provide conclusive evidence of the footprint of heparin binding to the capsid. However, in the case of foot-and-mouth disease virus (FMDV) this is not a technique that can be done easily and quickly. FMDV is an OIE controlled disease and any work with the virus or derivative thereof can only be performed in a biosafety level 3 (BSL-3) laboratory. Furthermore, the SAT serotypes have never been associated with the UK or Europe. The transport of SAT virus or inactivated antigen can only be performed under strict governmental guidelines. There are no cryo-electron microscopes around the world currently able to work with FMDV.

In this situation we believe the molecular docking simulation contributes necessary and important information to the final conclusions made from this research project. Both manual inspection and GOLD fitness score following heparin disaccharide docking to the SAT1 structure showed that the disaccharide fits much better to the mutant (GOLDscore: 127) containing positive charged residues at positions 111 and 112 of the VP1 protein, compared to the wild-type virus (GOLDscore: 102) with only one positive charge, at that position. The strongest evidence of interaction of heparin with the SAT1 or SAT2 capsids was observed with changes at VP1 residues 111 and 112. A similar fit was not observed for amino acid residue substitutions within the VP3 protein or other positions within the VP1 protein. We agree that ultimately experimental verification will be needed, however the present docking provides insight into the likely interaction and might help inform future experiments.

**Reviewer #2**: Symmetrical arrangement of positively charged residues around the 5-fold axes of SAT type foot-and-mouth disease virus enhances cell culture of field viruses.

FMD viruses of Southern African Territories (SAT) serotypes were adapted to growth on the BHK cell line. Adapted viruses gained the ability to enter cells by interaction with heparan sulphate proteoglycan (HSPG) via the selection of synonymous substitutions resulting in increased positive charge around the 5-fold symmetry axis of the viral capsid. The most important residues for adaptation were confirmed by characterising modified viruses generated by reverse genetics.

This is a meticulous study by well-respected authors which has generated robust data which adds some new information to the process of culture adaptation of FMDV.

**Reviewer #3**: The submitting groups here have a long background in FMDV and the manuscript is correspondingly authoritative. In the main the data hangs together well. The subject is the more problematic members of the FMDV serotypes, SAT1 and SAT2, generally regarded as difficult to grow and/or unstable. Selection of rapidly growing variants from the quasispecies by extensive passage results in isolates that bypass the integrin receptors and enter cells by the more generic mechanism of HS binding, as has been published for other serotypes. These viruses are characterized and reveal a predominance of charge changes with a subset of these clustered around the 5-fold axis. It is assumed the avidity of the cluster accounts for the new binding properties and to an extent this is confirmed by substitutions in molecular clones which indicate one (at 111-112 in VP1), sometimes with other surface exposed changes, are the key charged required. The knock-on of this is that field isolates required to be grown for vaccine use could be “fixed” by the introduction of the key change before being scaled up. This is useful but I did get the sense the story had been decided before the data were put together and a few more caveats might be wise. For example:

I lost the connection, if there is one, with the mutation and plaque morphology. While size and opacity relate to virus yield they are also affected by cpe and cell to cell transmission. There is still quite a range of titres in the adapted viruses. Are we sure it's all just better entry=better yield?

**Author’s comment**: The association between FMDV plaque formation or morphology have been initiated by Jackson *et al.*, (1996) who described that the efficient infection of cultured cells by a serotype O FMDV requires binding to heparan sulfate. Sa-Carvalho et al (1997) reported that adaptation of FMDV to BHK-21 cultured cells requires binding to heparin and selects for a small, clear-plaque virus that replicates in BHK-21 and CHO-K1 cells. In contrast, the large, turbid-plaque variant of the virus did not replicate in CHO-K1 cells. An amino acid residue in the VP3 protein of FMDV was linked to the small, clear-plaque viral phenotype and ability to grow in CHO-K1 cells. The phenotypic change of viruses adapted to cultured cells is not unique to FMDV and has been observed for many other viruses. As an example, Byrnes and Griffin (2000) reported on a large-plaque mutant of the Sindbis virus that showed reduced binding to heparan sulfate in culture and heightened viremia following animal challenge. More recently McLeish et al., 2012 reported that a cluster of positively charged residues on the surface of enteroviruses are marked by a reduction in plaque sizes and ability to bind to sulphated compounds.

We have made the link between the selection of small, clear-plaques of SAT1 and SAT2 viruses, following serial passage on BHK-21 cells in Table 1 and the descriptive text in lines 120-160. This phenotype was also linked with the ability to infect CHO-K1 cells, that do not express the integrin receptors used by FMDV for cell entry, and prevention of cell entry by heparin.

I think the exceptions illustrated by 158K and 83K in VP1 and 158K in VP3 need a little more weight. They are inhibited by heparin but do not go in BHKs. There is a trend in the manuscript to the view that +ve charge=HS binding=enhanced entry=enhanced yield. But these mutants do not fit - why not? What’s not right about them?

**Author’s comment**: We agree with the reviewer regarding the exceptions observed and have stated in lines 377-384 of the discussion that none of the following mutants, 158K and 83K in VP1 and 158K in VP3, showed any growth advantage on BHK-21 cells, compared to the wild-type chimeric vSAUSAT2 virus. We also concluded that our results suggest that KRR residues at positions 110-112 of VP1 are the most important residues for cell culture adaptation and expansion of cell tropism for SAT1 and SAT2 viruses. Also the abstract states that only positively charged residues at position 110-112 of VP1 enhanced infectivity of BHK-21 cells. We improved on the clarity of this conclusion by adding the following statement in the concluding paragraph of the discussion: “*We found that a cluster of positively charged residues at positions 110-112 of VP1, are symmetrically arranged around the 5-fold axis of the virion and are the most important residues for cell culture adaptation and expansion of cell tropism for SAT1 and SAT2 viruses.*”

Cell entry by means of heparin sulfate is probably more complex than the equation provided above. However, a symmetrically arranged positive charged amino acid cluster does provide a growth advantage in cultured cells, as evidenced by the results in our study. This has also been observed for enteroviruses (McLeish et al., 2012).

I think the application should be more reserved. Only two of the final mutants reach titres (>10^8) that would be respectable enough for mass culture and it remains to be seen how many field isolates would do the same when engineered in this way.

**Author’s comment**: We have added the following statement in the concluding paragraph of the discussion: “*We found that a cluster of positive charged residues at positions 110-112 of VP1, are symmetrically arranged around the 5-fold axis of the virion and are the most important residues for cell culture adaptation and expansion of cell tropism for SAT1 and SAT2 viruses.*” In the abstract it states that only positively charged residues at position 110-112 of VP1 enhanced infectivity of BHK-21 cells.

It seems probable that the mutants here would generate the same antibody response on immunisation at the WT but it is not yet proven. It's possible that they could affect antigen processing.

**Author’s comment**: A vaccine producer needs to be particularly vigilant that propagation does not bias the antigenic profile of their vaccine master seed viruses. A small probability may exist that the amino acid substitutions performed here, may have an effect on the antigenic properties of the virus; however, we have performed limited antigenic matching studies and did not find a significant change in antigenicity of the mutant viruses. We have explained this aspect in more detail in our comments to reviewer 1 in PART II and have made the appropriate changes to the text of the manuscript.

There are recombinant routes to FMDV vaccines, the rec. Adeno for example, where the need to culture is bypassed anyway. Would these mutations benefit or hinder such approaches?

**Author’s comment**: Live viral vector vaccines, like Adenovirus vector that deliver the capsid-coding and 3C-protease sequences of FMDV to cells in vaccinated animals and allow the expression and release of virus-like particles to the immune system will find little benefit with FMDV cell culture adaptation. There is no need for passage of FMDV through cell culture and therefore synthesis of VLPs do not need the symmetrical clusters of positive charge residues on the viral capsid surface. However, these technologies, although excellent approaches in FMD-free regions of the world, is still far from effective control of FMD in the endemic regions, like Africa and Asia.

- **Part II – Major Issues: Key Experiments Required for Acceptance**

**Reviewer #1**: No major issues have been found. However, the reviewer suggests the authors to add discussions about whether making those chimeric viruses with positive charged residues clustered on the 5-fold vertices might alter the antigen properties of the viruses. The animal might develop immune response to those areas which might defeat the purposes of vaccine development of emerging field strains.

**Author’s comment**: This is a valid point. The evidence that the modification at the five-fold axis did not significantly alter the antigenic properties of the viruses come from two independent experiments. In the first experiment we have subjected the v^SAU^SAT2 intra-serotype chimeric virus and the mutant viruses to virus neutralization tests (VNT) using SAT2/SAU/6/00 vaccinated cattle sera and calculated the cross-reactivity (r1-values) as recommended in the OIE Manual (2017) for vaccine matching studies. The cross-reactivity of the v^SAU^SAT2 and the mutant viruses were not only comparable to each other but also to the original SAT2/SAU/6/00 virus calf thyroid (CT) isolate. Similarly, the SAT1-SAT2 chimeric virus and its mutants were tested against available SAT1 vaccinated cattle sera and the cross-reactivity were comparable. In the second experiment cattle were vaccinated with a vaccine prepared from the SAT2/SAU/6/00 that was passaged 58 times. The serum used in the VNT neutralize both the original isolate (SAU/6/00^CT1^) and SAU/6/00^BHK58^ equally well (r_1_-value=1). The data indicated that the antigenic properties of serially passaged virus or the mutant viruses were not significantly different. Our data is in agreement with the observations by Zhao et al 2003 (J Virol.) that amino acid substitutions in cell culture adapted serotype O viruses did not significantly change the antigenic character of the virus.

We have added more information in the results, discussion and methods sections (lines 257-266, 405-411 and 498-503) to better explain the observations made during cross-reactivity studies.

**Reviewer #2**: This is a meticulous study by well-respected authors which has generated robust data which adds some new information to the process of culture adaptation of FMDV. However, the main claims of the study are already very well established. References (cited in the manuscript) 21, 44, 45 and 23, all describe previous culture adaptation of FMDV involving acquisition of positive charge around the five-fold axis. Therefore, the novelty of the current study is not what would normally be consistent with publication in PLoS Pathogens.

What is of more concern is that the introduction and discussion does not make this situation clear. Despite the references being cited within the paper, there is no explanation in the introduction, of these previous studies (e.g. references 21, 44, 45, 23) having already identified acquisition of positive charge around the 5-fold as a mechanism of culture adaptation. This seems bizarre. Instead the following statement is included:

“The genetic alterations associated with increased cell killing activity during cytolytic passages of the SAT serotype FMD viruses in BHK-21 cells are largely unknown. Nevertheless, it has been noted that amino acid substitutions accumulate in their capsids during serial passaging (44–46)”

One of the references at the end of this statement is even from one of the authors of the current study and specifically describes the acquisition of positive charge around the five-fold as a mechanism of culture adaptation of SAT type viruses. In the context of the novel claims of the current manuscript this statement and other omissions appear misleading. Perhaps hopefully all of this is the result of lack of oversight, but I’m afraid it is rather disappointing.

**Author’s comment**: We have removed the paragraph in the introduction that was seen as a concern and replaced it with a much more detailed explanation of HS binding positions observed for viruses from different serotypes. We believe the information provides a clearer and comprehensive picture of the known amino acid residues and positions that accumulate during serial passage of different viruses, and shortcomings in our knowledge of the mechanisms of cell culture adaptation.

- **Part III – Minor Issues: Editorial and Data Presentation Modifications**

**Reviewer #1**:

1. The paper still contains some typos that need to be read carefully and correct. Eg. In Ln177 “the roadmap programme”, the Roadmap should be capitalized to be consistent with Ln534. Another example in Ln263 “heparinise” should be “heparinase”?

**Author’s comment:** We have made the corrections. The “Roadmap” has been corrected to RIVEM throughout the document. Also, the spelling of “heparinase” has been corrected throughout document.

2. The authors need to check all the references carefully. Some of the references are not correctly cited. For example, for the same Ln177 mentioned above. Reference 48 is not the Roadmap program but the program GRID for docking as shown in Ln191. Again, on Ln534, the Roadmap was cited as reference 47, which is a very old review paper for virus-polysaccharide interactions. None of the Roadmap was cited properly. The reviewer does not have time to check all the references for the authors.

**Author’s comment:**	We have checked and corrected the references.

3.	In order to improve the readability, instead of writing the mutagenesis primer sequences in the text (Ln455 to 463), the reviewer suggests the authors to list them in a table.

**Author’s comment**: 2. We have added a Table 4 to summarize the mutagenesis and In-Fusion cloning primers and remove the primer sequences from the text to improve readability.

**Reviewer #2**: It was not clear what significance is provided by the in-silico docking analysis. Also it is only referred to as GRID, a non-expert introduction to what this means and how it works would be useful. I found the description and nomenclature of the engineered viruses was difficult to follow.

**Author’s comment:** Regarding the in-silico docking and the GRID, this has now been clarified in the text.

The description and the nomenclature of the engineered viruses has been re-worked a number of times and we found that what we have used in this manuscript was the best way to describe the mutant viruses and also help the reader understand which mutation is referred to with each engineered virus.

_____________________________________
