# Supplementary material for: Symmetrical arrangement of positively charged residues around the 5-fold axes of SAT type foot-and-mouth disease virus enhances cell culture of field viruses
Source: PLoS Pathog. 2020 Sep 29;16(9):e1008828. doi: 10.1371/journal.ppat.1008828 (PMC7577442; doi:10.1371/journal.ppat.1008828)
Supplement: Attachment — Submitted filename: Rebuttal letter PLOS_Path 2revision_July 2020.docx [file ppat.1008828.s004.docx]

**Agricultural Research Council**

**Onderstepoort Veterinary Research Institute**

**Transboundary Animal Diseases Programme**

**Old Soutpan Road**

**Onderstepoort, 0110**

**South Africa**

**Tel. +27 12 529 9575**

**Fax. +27 12 529 9505**

14 July 2020

To: The Associate Editor, Section Editor and Editor-in-Chiefs– PLoS Pathogens Journal

**Ref: PPATHOGENS-D-20-00242 -** “**Symmetrical arrangement of positively charged residues around the 5-fold axes of SAT type foot-and-mouth disease virus enhances cell culture of field viruses”**

Thank you for communicating to us the expert reviewer’s comments of our manuscript by Chitray et al., with reference number PPATHOGENS-D-20-00242. All authors are in agreement with the revised manuscript.

We agree with the Editor that we may not have addressed the one aspect from reviewer 2 comments during the first round of corrections. We found the comment from the Editor very useful, and we have now addressed all of the comments from the reviewer 2 accordingly (point-by-point list enclosed). The second reviewers did state “this is a meticulous study by well-respected authors which has generated robust data”, and for that we are thankful. We have revised the manuscript to include additions to the text to improve the shortcomings. The changes are tracked in the enclosed revised document.

We hope that the revised manuscript may now be acceptable for publication in the PLoS Pathogens Journal.

Sincerely yours,

Francois F Maree, PhD

Specialist Scientist

Agricultural Research Council

Onderstepoort Veterinary Research Institute

Vaccines and Diagnostic Development

**Response to Editor**

Editor’s comment: Thank you very much for submitting your manuscript "Symmetrical arrangement of positively charged residues around the 5-fold axes of SAT type foot-and-mouth disease virus enhances cell culture of field viruses" for consideration at PLOS Pathogens. As with all papers reviewed by the journal, your manuscript was reviewed by members of the editorial board and by several independent reviewers. The reviewers appreciated the attention to an important topic. Based on the reviews, we are likely to accept this manuscript for publication, providing that you modify the manuscript according to the review recommendations.

The authors have responded appropriately to most comments by the reviewers. However, Reviewer #2 had some specific questions regarding the novelty of the work but more specifically the way the authors referred to prior work and discussed this in the context of this manuscript; The comments in questions are:

Reviewer #2: This is a meticulous study by well-respected authors which has generated robust data which adds some new information to the process of culture adaptation of FMDV. However, the main claims of the study are already very well established. References (cited in the manuscript) 21, 44, 45 and 23, all describe previous culture adaptation of FMDV involving acquisition of positive charge around the five-fold axis. Therefore, the novelty of the current study is not what would normally be consistent with publication in PLoS Pathogens.
What is of more concern is that the introduction and discussion does not make this situation clear. Despite the references being cited within the paper, there is no explanation in the introduction, of these previous studies (e.g. references 21, 44, 45, 23) having already identified acquisition of positive charge around the 5-fold as a mechanism of culture adaptation. This seems bizarre. Instead the following statement is included:
“The genetic alterations associated with increased cell killing activity during cytolytic passages of the SAT serotype FMD viruses in BHK-21 cells are largely unknown. Nevertheless, it has been noted that amino acid substitutions accumulate in their capsids during serial passaging (44–46)”
One of the references at the end of this statement is even from one of the authors of the current study and specifically describes the acquisition of positive charge around the five-fold as a mechanism of culture adaptation of SAT type viruses. In the context of the novel claims of the current manuscript this statement and other omissions appear misleading. Perhaps hopefully all of this is the result of lack of oversight, but I’m afraid it is rather disappointing.

The authors have removed the statement that is mentioned but they have not addressed the central concern of the reviewer. They need to be clear on the response about these concerns.

**Author’s response:**

We have interpreted the reviewer 2 comment as having three parts, (1) a statement in the introduction that might be interpreted as misleading; (2) a more detailed explanation in the introduction is needed to describe the acquisition of positive charge during cell culture adaptation of FMDV; and (3) the novelty of the study. In the new version of the manuscript, each of the comments by reviewer 2 were considered carefully and addressed as follows:

1. We have removed the problematic statement that mentioned, “The genetic alterations associated with increased cell killing activity during cytolytic passages of the SAT serotype FMD viruses in BHK-21 cells are largely unknown.”
2. To address the comment by the reviewer that no explanation were provided in the introduction, of these previous studies having already identified acquisition of positive charge around the 5-fold as a mechanism of culture adaptation, we have rewritten the paragraph in the introduction. The new paragraph describe all the previous findings in serotype O and A viruses regarding genetic changes that was observed during cell culture adaptation. A similar change to serotype A, associated with cell culture adaptation was seen for a SAT1 virus, therefore we included the statement “*Cell culture adapted serotype A and SAT1 viruses have also been reported to use cell receptors other than integrins to gain entry into cells.*” (lines 109-111).

However, in this study although positive charges were observed during cell culture adaptation of the SAT1 virus, we were unable to proof in our previous study Maree et al. (2011) that the adaptation was due to interaction with heparin sulphate proteoglycan molecules on the cell surface. Thus, we noted “*However, the interaction of these residues with cellular receptors is unknown.*” (line 114-115).

It is clear from the different studies that genetic changes for viruses from different serotypes and even within a serotype can be vastly different. No single amino acid residue substitution could explain the molecular mechanism of cell culture adaptation in all the FMD viruses.

3. The gap in our understanding of the interaction of SAT capsids with HS and a possible mechanism for cell entry in cultured cells was addressed by adding a statement at the end of paragraph 5, saying “*The molecular basis of the interaction of SAT viruses with HS and the mechanism of cell entry in cultured cells is still elusive.*” (lines 126-127) We believe the novelty of the work describe in this manuscript is that we provided proof from (i) structural studies to show the interaction of negatively charged sulphated heparin molecules can interact with the symmetrically arranged positive clusters on the 5-fold axis; and (ii) mutational and HS inhibition studies. In lines 140-142 we added a statement “*Here, we provide the structural basis for cell culture adaptation of SAT1 and SAT2 viruses and showed the interaction of the capsids with HS to allow the effective entry into cultured cells.*” We also mentioned that five mutant viruses, containing novel HS-binding sites, indeed interact with HS. One such mutation were able to interact with HS and enhanced infectivity and virus titre in BHK-21 cells. This for the first time provide a mechanism universal for SAT1 and SAT2 viruses to enter cultured cells via an alternative receptor.

We do believe that the changes made to the text indeed improve clarity on the novelty of the study and also its importance in the FMDV and broader virology disciplines.

_____________________________________
